# Low Complement Factor H-Related 3 (CFHR3) Expression Indicates Poor Prognosis and Immune Regulation in Cholangiocarcinoma

**DOI:** 10.1155/2022/1752827

**Published:** 2022-09-30

**Authors:** Haoran Wang, Meng He, Zheng Zhang, Wenze Yin, Bixuan Ren, Yujia Lin

**Affiliations:** Department of Hepatobiliary Surgery, Second Affiliated Hospital of Harbin Medical University, Harbin, Heilongjiang, China

## Abstract

**Background:**

Cholangiocarcinoma (CCA) is a cancerous tumor that leads to a high rate of morbidity and death. Complement factor H-related 3 (CFHR3) is a gene belonging to the CFHR gene family. In this study, we investigated the usefulness of CFHR3 in the diagnostic stage and CCA prognosis prediction. In the interim, we looked at its coexpressed genes and their roles. The correlation between CFHR3 and immunological infiltration was also investigated.

**Methods:**

The expression of the genes data and the clinical information were obtained from the databases of The Cancer Genome Atlas (TCGA) together with the Gene Expression Omnibus (GEO). The crucial gene was found to be the overlapping gene in the two databases. The area under the curve (AUC) and the Kaplan-Meier survival curve were used to describe the usefulness of the predictive prognosis of CCA patients. Univariate regression analysis and multivariate survival analysis were performed to find the independent prognosis factors. The PPI network was constructed based on the STRING database, and the coexpression approach was utilized in predicting the coexpression genes. Gene Ontology (GO) and Kyoto Encyclopedia of Genes and Genomes (KEGG) enrichment analyses were also performed to identify the related functions. Additionally, the probable mechanism of the important gene was examined using gene set enrichment analysis (GSEA). The correlation between CFHR3 and immune infiltration was discovered using TIMER. The LncACTdb 3.0 database was used to analyze the location of CFHR3 in the cell. The cBioPortal database was used to find the mutation in CFHR3.

**Results:**

TCGA datasets and GEO datasets revealed an elevated expression level of CFHR3 in normal tissues as well as a lower expression level in cholangiocarcinoma tissues in the present research. The low expression level of CFHR3 was related to an unfavorable prognosis. Using CFHR3 expression in diagnosis and predicting the patient prognosis (AUC = 1.000) is valuable. Using the CFHR3 gene and a time-lapse prediction, we could estimate survival rates over 1, 2, and 3 years. The AUC values were more than 0.6(AUC = 0.808; 0.760; 0.711). Functional enrichment analysis revealed a substantial correlation between this signature and complement and coagulation cascades. The same outcomes from GSEA were achieved. We found the key gene widely exists in the nucleus, exosomes, and cytoplasm of normal cells using the LncACTdb 3.0 database. In immune regulation analysis, we identified that the expression level of CFHR3 had a positive correlation with infiltrating levels of B cells, neutrophils, and macrophages, but correlated negatively with cholangiocarcinoma cells, CD8+ T cells, and monocytes.

## 1. Introduction

Cholangiocarcinoma (CCA) is the second most common tumor found in the liver. CAA is characterized as such by originating from the biliary system [1]. The incidence and mortality rates of cholangiocarcinoma are increasing year by year all over the world [2]. The primary method for treating tumors is surgical resection, however, patients frequently miss the best window for surgery and pass away because the tumor is discovered at an advanced stage [3]. Therefore, it is important to screen certain valuable genes for a more efficient prognosis prediction and to provide optimal customized treatment.

Recent studies have shown that some genes, such as LIMA1, HDAC1, ITGA3, ACTR3, GSK3B, ITGA2, THOC2, PTGES3, HEATR1, and ILF2, are associated with the prognosis of patients with cholangiocarcinoma [4]. However, there is still an urgent need to identify more genes to obtain more accurate predictions. CFHR3 belongs to a gene family that also consists of CFHR1, CFHR2, CFHR4, and CFHR5. A collection of complement proteins with these genes are closely related [5]. According to reports, CFHR3 may be a potential biomarker for the disease hepatocellular carcinoma (HCC) [6]; however, as the second highest type of cancer in the liver, the correlation between CFHR3 expression and its clinical significance of CCA remains unclear.

Here, we identified CFHR3 as a key gene and hypothesized that CFHR3 has a correlation with prognosis and immune regulation of cholangiocarcinoma. Bioinformatics was used to assess this theory. To better understand CFHR3 function, we also looked into the coexpression genes and the protein-protein interaction (PPI) network. Immune infiltration was also explored to confirm that the expression of CFHR3 correlates with immune regulation. Finally, we performed a further investigation of the molecular mechanism of CFHR3. CFHR3 might be employed as a marker in predicting immune and prognosis-related status in patients with CCA.

The paper's organization paragraph is as follows: the materials and methods is presented in Section 2.  Section 3 discusses the experiments and results.  Section 4 analyzes the discussion of the proposed work. Finally, in Section 5, the research work is concluded.

## 2. Materials and Methods

### 2.1. Data Obtaining

The TCGA database (https://portal.gdc.cancer.gov/) was utilized in evaluating the CFHR3 expression. Other datasets, including GSE40367, GSE31370, and GSE32879 [7–9], were collected from the GEO database (https://www.ncbi.nlm.nih.gov/geo/) and utilized to examine CFHR3 expression and further validate our findings.

### 2.2. Differential Analysis of the Key Gene

In the four datasets, differentially expressed genes were evaluated with the aid of the online tools UCSC Xena (https://xena.ucsc.edu/) [10] together with GEO2R (https://www.ncbi.nlm.nih.gov/geo/geo2r/) with the condition used being adjusted *p* value < 0.05 coupled with |log2 fold change (FC)| > 1.

Using univariate regression analysis, the survival-related genes in the TCGA database were initially identified. In order to give further clinical details, the Genotype-Tissue Expression Project (GTEx) database was also utilized. The overlapping gene was selected and illustrated using the “Venn” package [11]. The volcano maps and box plots were completed using ggplot2 package R software and GEO2R online tools to illustrate the differential appearance.

### 2.3. Survival Analysis

The TCGA database provided the data necessary for the survival analysis. The Kaplan–Meier curves were constructed with the aid of GEPIA (http://gepia.cancer-pku.cn/index.html) [12].

The Kaplan-Meier curves were used to compare the differences reported in the OS and DFS. We were thorough in our evaluation and got rid of some information that did not match the requirements. Patients were classified into two groups according to their CFHR3 expression levels, namely, the high- and low-CFHR3 expression groups. This data was used to build the baseline data table and to perform both the univariate and multivariate regression analyses. To confirm the key gene's accuracy as a prognostic molecule, ROC curves of patient diagnosis were generated using R packages pROC and ggplot2.

### 2.4. Enrichment Analysis and Construction of PPI Network

CFHR3-related genes were screened with the use of STRING (http://string.embl.de/) [13].

The medium confidence rate > 0.4 was regarded as significant. The enrichment analysis was conducted with the aid of DAVID (https://david.ncifcrf.gov/) [14]. Gene ontology (GO) analysis and Kyoto Encyclopedia of Genes and Genomes (KEGGs) pathway analyses are the two types of enrichment analysis for the key gene. The criterion was fixed at *p* < 0.05. ggplot2 package and R software were used to complete visualization. GSEA software (http://software.broadinstitute.org/gsea/index.jsp) was utilized in performing the gene set enrichment analysis [15].

### 2.5. Coexpression Gene Screening and Functional Annotation

To determine the coexpression relationship, Pearson's correlation coefficients were computed between the main gene and other genes. We selected genes having a |Pearson′s correlation coefficient| > 0.5 as well as *p* value < 0.05. We selected the top15 lncRNA, mRNA, and all miRNAs to create a heat map using R software. Alluvial plotting was performed to show the associations between these genes. The functional annotation of the genes was completed with the aid of Metascape (http://metascape.org/gp/index.html#/main/step1) [16].

### 2.6. Immune Cell Infiltration Analysis

To examine the expression profiles of several immune cells, we utilized the Human Protein Atlas (HPA, https://www.proteinatlas.org/). TIMER (https://cistrome.shinyapps.io/timer/) was utilized to investigate the relationship between the expression of CFHR3 and immune cell infiltration and immune cell biomarkers in cholangiocarcinoma [17, 18].

### 2.7. CFHR3 Genetic Location and Alteration Analysis

The position of CFHR3 in the cell was analyzed using LncACTdb 3.0 database (http://www.bio-bigdata.net/LncACTdb/) [19]. The cBioPortal database (http://www.cbioportal.org/) was used to show the key gene alteration [20, 21].

### 2.8. Statistical Analysis

R software and its resource packages were used for statistical analysis and to create related visualization graphics. A Wilcoxon rank-sum test or Student's *t*-test was used to calculate the difference in expression between normal and cholangiocarcinoma tissues. The relationship between other genes and CFHR3 was determined using Pearson's correlation.

To determine the significance of the difference among the survival curves, Kaplan-Meier plots were plotted and log-rank tests were conducted. Statistically significant differences were defined as those with a value of *p* < 0.05. For all statistical tests in this passage, *p* < 0.05 was set as the criterion of the statistical significance.

## 3. Results

### 3.1. Key Gene Identified and Differential Expression Analysis

**Figure 1:**
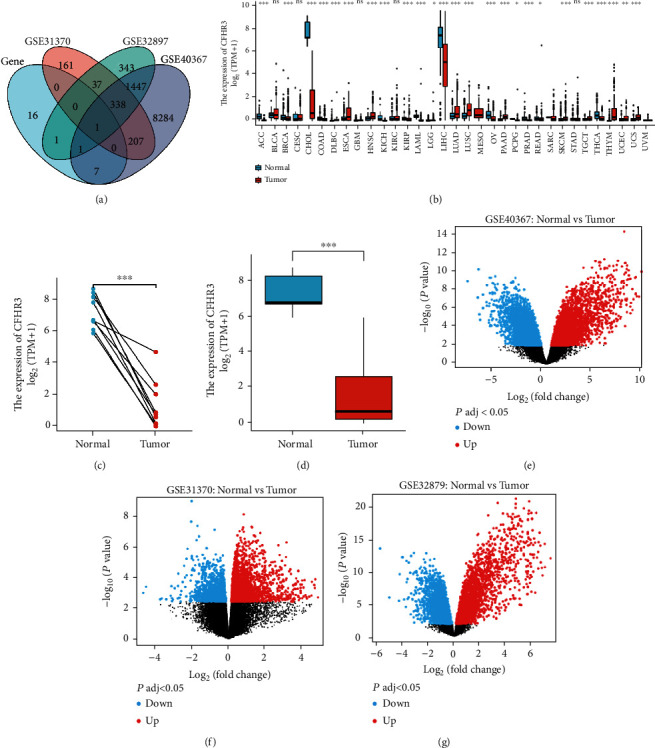
The difference expression of CFHR3 in CCA. (a) A Venn diagram of intersection of genes related with prognosis from the TCGA and GSE40367, GSE31370, and GSE32879. (b) The expressions of CFHR3 in common tumors. (c) A line diagram of the difference expression of CFHR3 in CCA. (d) A box plot of the difference expression of CFHR3 in CCA. (e–g) The volcano maps of CFHR3 in GSE40367, GSE31370, and GSE32879.

Data was collected from TCGA and GEO datasets. We completed the differential analysis and preliminary univariate regression analysis. Twenty-six (26) genes were selected as target genes (Table 1). Upon examination, we found three datasets, the GSE40367, GSE31370, and GSE32879, which contained the cholangiocarcinoma information and normal information. Differential analysis was performed to find the target genes. Finally, the overlapping gene was screened by the Venn diagram as our key gene (Figure 1(a)).

We did a series of differential expression analyses after identifying the crucial gene, CFHR3. We first discovered a difference in expression between cholangiocarcinoma and other malignancies. The expression of CFHR3 varies widely across 21 cancer types (Figure 1(b)). A higher expression of CFHR3 in normal tissues and a lower expression in cholangiocarcinoma tissues was observed in TCGA datasets (Figures 1(c) and 1(d)). Low expression of CFHR3 in CCA was observed in GSE40367, GSE31370, and GSE32879 based on the GEO database data (Figures 1(e)–1(g)). This data demonstrates that CFHR3 expression differs between normal tissues and cholangiocarcinoma tissues.

### 3.2. Correlation between Clinical Features and CFHR3 Expression of Cholangiocarcinoma

**Figure 2:**
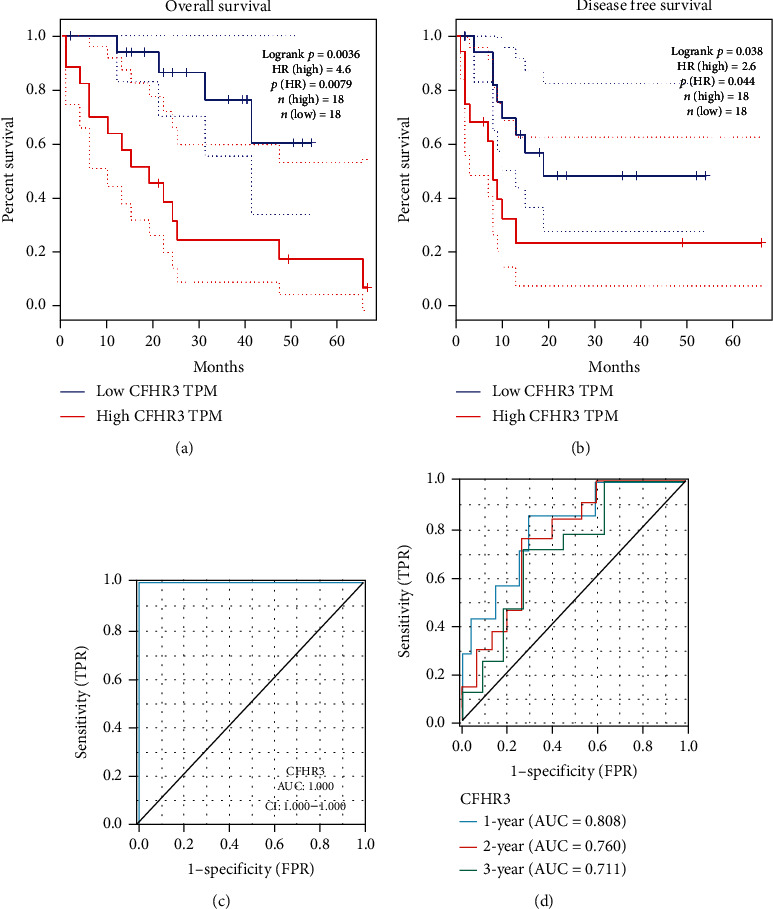
The value of CFHR3 in predicting the prognosis. (a) The OS survival curves comparing patients with high (red) and low (blue) CFHR3 expression in CCA (*p* < 0.05) (b) The DFS survival curves comparing patients with high (red) and low (blue) CFHR3 expression in CCA (*p* < 0.05). (c) The ROC curve to confirm accurate value of CFHR3 expression in diagnosis and predicting prognosis (*AUC* = 1.000) (d) Time-dependent survival ROC curve of CFHR3 to predict 1-, 2-, and 3-year survival rates. All AUC values were above 0.6(AUC = 0.808; 0.760; 0.711).

We used GEPIA to create Kaplan-Meier survival curves to evaluate the relationship between clinical prognosis and the main gene. As the curves shown, cholangiocarcinoma patients with lower CFHR3 expression showed a lower OS (log-rank *p* = 0.0036) and a poorer DFS (log-rank *p* = 0.038). The low expression level of CFHR3 is related to an unfavorable prognosis. (Figures 2(a) and 2(b)).

The ROC curve was used to confirm accurate values of CFHR3 expression in diagnosis and prognosis prediction (AUC = 1.000) (Figure 2(c)). To predict the survival rates over 1, 2, and 3 years, the time-dependent survival ROC curve of CFHR3 was generated. AUC values were all more than 0.6(AUC = 0.808; 0.760; 0.711) (Figure 2(d)). All of these results suggest that our key gene has an effective prognostic value.

The clinical data was gathered from the TCGA database and utilized to screen for the independent prognostic factor. Variables including age, gender, TNM stages, pathology stage, histological type, CA199 level, vascular invasion, and perineural invasion were included.

These results are shown in the baseline information table (Table 2). Next, we completed both univariate cox analysis and multivariate cox analysis (Table 3). Consequently, the perineural invasion was identified as an independent prognostic factor (*p* < 0.05).

### 3.3. PPI Network Construction and Underlying Function Analysis of CFHR3

**Figure 3:**
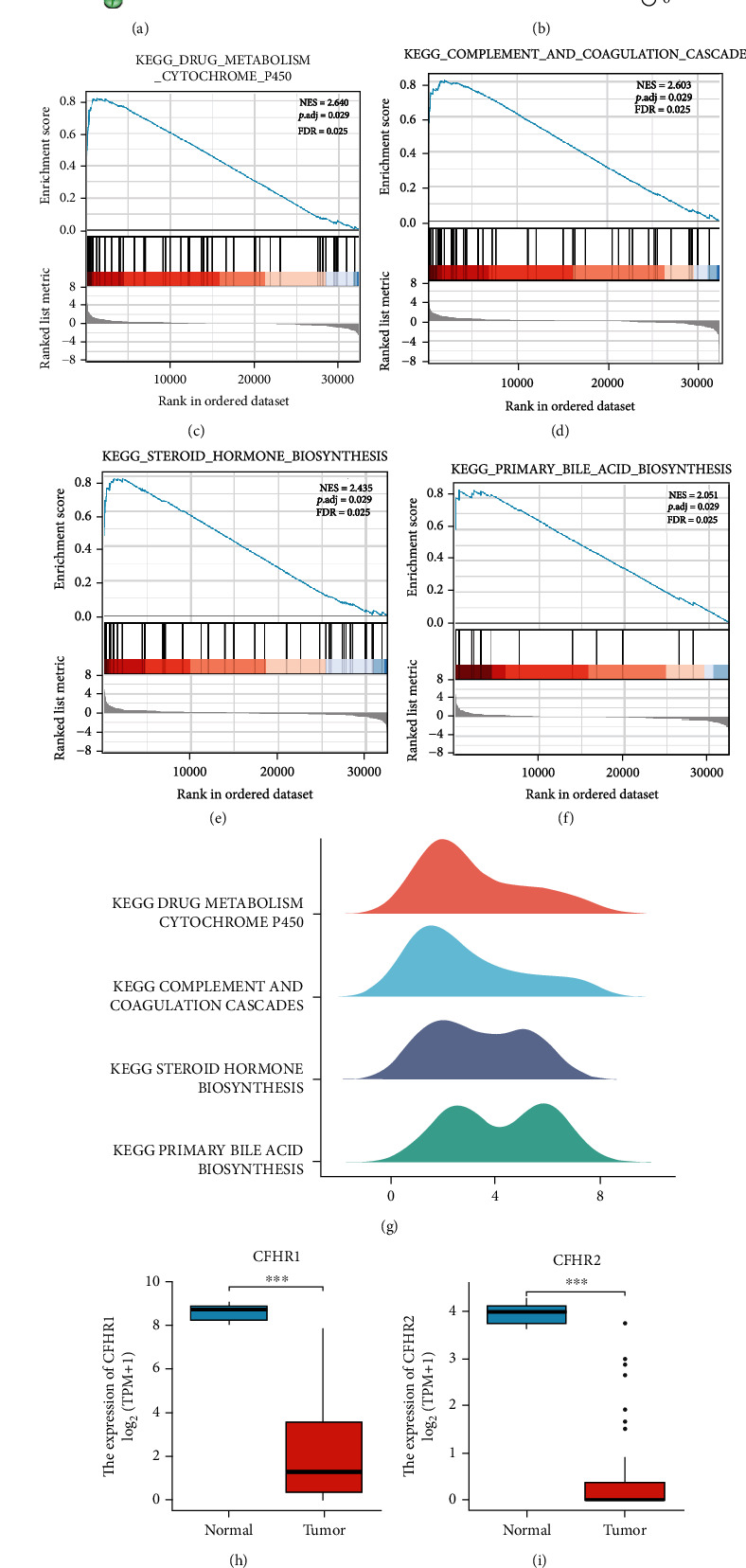
The PPI network and GO, KEGG analysis. (a) PPI network of CFHR3 in STRING. (b) GO and KEGG enrichment of interacted genes of CFHR3. (c–g) GSEA enrichment analysis of CFHR3. (h-k) The expression of CFHR1, CFHR2, CFHR4, and CFHR5.

Ten (10) genes were screened for CFHR3-related genes with remarkable interaction, including CFHR1, CFH, CF8, CFI, C3, NIPA2, MNS1, NIPA1, TUBGCP5, and CYFIP1. With the aid of the STRING database, we carried out the PPI network analysis of CFHR3 and CFHR3-related genes (Figure 3(a)).

The key gene and its corresponding genes were strongly enriched in the BP category, which included regulation of humoral immune response, regulation of complement activation, and regulation of protein activation cascade, according to the GO analysis.

In the CC category, there was an enrichment of genes in blood microparticles, mRNA cap-binding complex, as well as dendrite terminus.

Magnesium ion transmembrane transporter activity, serine-type endopeptidase activity, and serine-type peptidase activity were all enriched in the MF category.

Results recorded from the KEGG pathway analysis indicated that the enrichment of genes was primarily in two pathways, namely, complement and coagulation cascades, and staphylococcus aureus infection. (Figure 3(b)).

We also analyzed the GSEA results of the TCGA database. As the maps show, the CFHR3 expression group was enriched in the drug metabolism cytochrome P450, complement and coagulation cascades, steroid hormone biosynthesis, and primary bile acid biosynthesis (Figures 3(c)–3(g)).

Finally, we observed CFHR1 in the PPI network, which is a member of the CFHR gene family; therefore, we performed the differential analysis of the CFHR gene family in cholangiocarcinoma. It was surprising that the data showed that all of the genes in this gene family had low expression in tumor tissue and high expression in normal tissues (Figures 3(h)–3(k)).

### 3.4. Coexpression Molecular Analysis of CFHR3 and Functional Annotation

**Figure 4:**
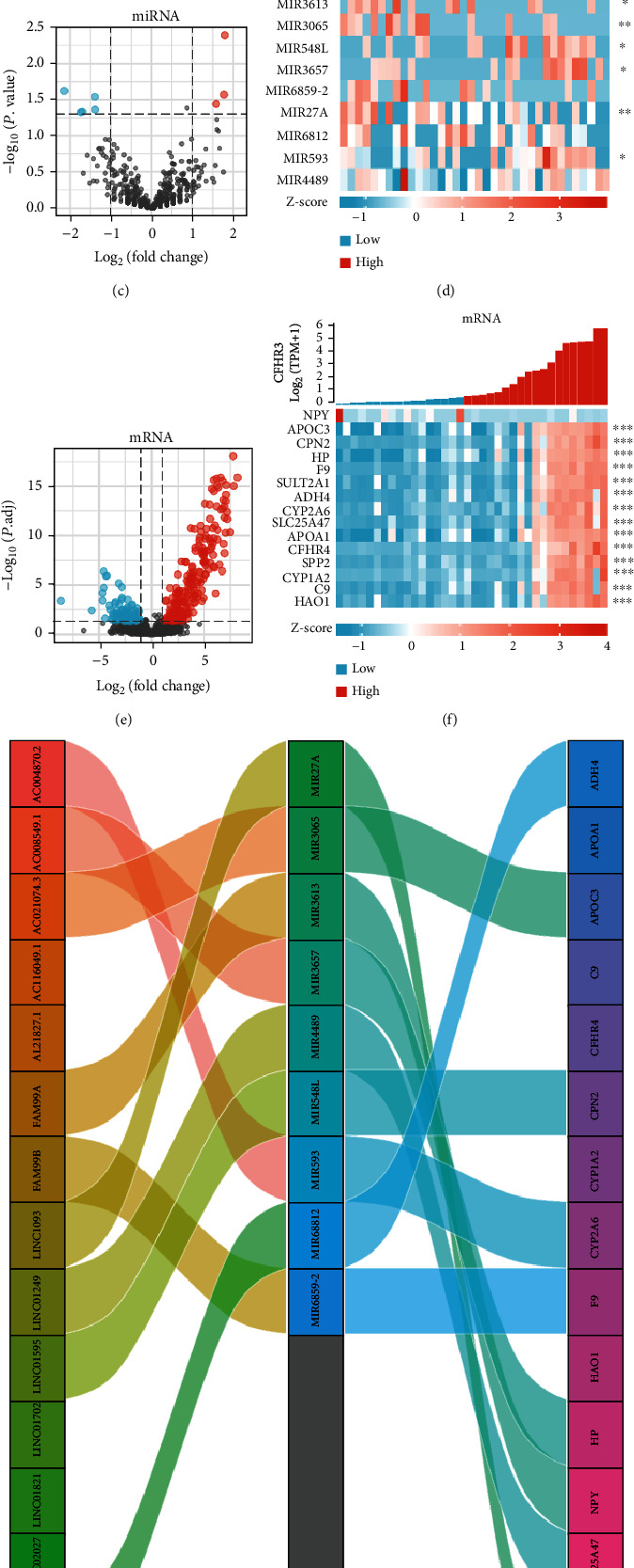
The coexpression genes analysis. The volcano map (a) and the heat map (b) of DElncRNAs. The volcano map (c) and the heat map (d) of DEmiRNAs. The volcano map (e) and the heat map (f) of DEmRNAs. (g) A Sankey diagram to show the relationship of the DElncRNAS, DEmiRNAs, and DEmRNAS. (h) The functional annotation of these genes in Metascape.

The coexpression method was used to predict the correlations among DElncRNAs, DEmiRNAs, and DEmRNAs with CFHR3 expression in patients with cholangiocarcinoma. The differential expression found in lncRNAs, miRNAs, and mRNAs is shown in the volcano maps and heat maps (Figures 4(a)–4(f)). The interrelationships between these genes are also illustrated in Figure 4(g).

Both GO and KEGG analyses showed that the functions of these genes were highly enriched in lipid catabolic process, monocarboxylic acid metabolic process, regulation of complement cascade, and gene silencing by miRNA (Figure 4(h)).

### 3.5. CFHR3 Is Associated with Immune Infiltration

The Human Protein Atlas (HPA) and TIMER database were utilized for additional investigation on the correlation between tumor immune microenvironment and genes.

**Figure 5:**
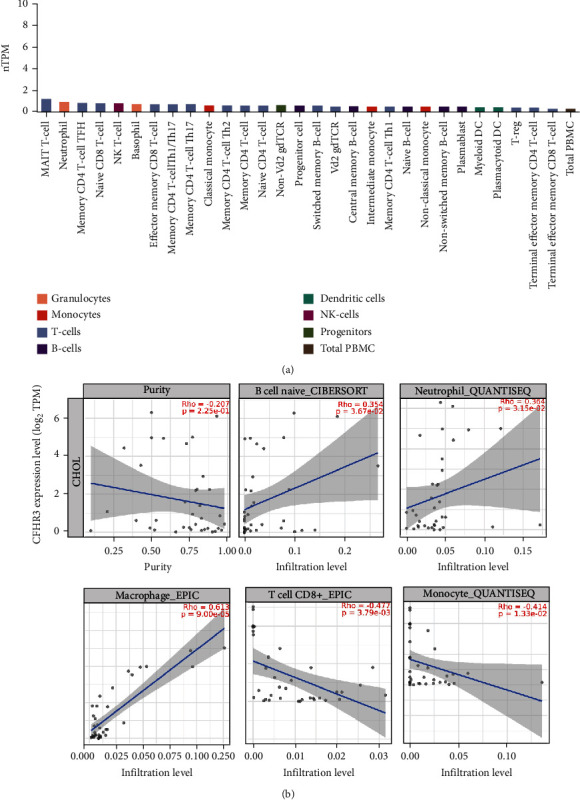
The immune infiltration analysis results. (a) The expression of immune cells in HPA. (b) The relationship between immune cells and CFHR3.

The HPA database was used to determine the expression of eight (8) different types of immune cells: granulocytes, monocytes, T cells, B cells, dendritic cells, NK cells, progenitors, and total peripheral blood mononuclear cells (PBMCs). To investigate the relationship between immune cells and CFHR3, TIMER was utilized (Figure 5(a)). The results were as follows; the expression level of CFHR3 had a positive relationship with the infiltrating levels of B cells (*r* = 0.354, *p* = 3.67*e* − 02), neutrophils (*r* = 0.364, *p* = 3.15*e* − 02), macrophages (*r* = 0.613, *p* = 9.00*e* − 05), but negatively correlated with tumor purity (*r* = −0.207, *p* = 2.25*e* − 01), CD8+ T cell (*r* = −0.477, *p* = 3.79*e* − 03), and monocytes (*r* = −0.414, *p* = 1.33*e* − 02) (Figure 5(b)).

### 3.6. CFHR3 Genetic Location and Alteration Analysis in Patients with Cholangiocarcinoma

**Figure 6:**
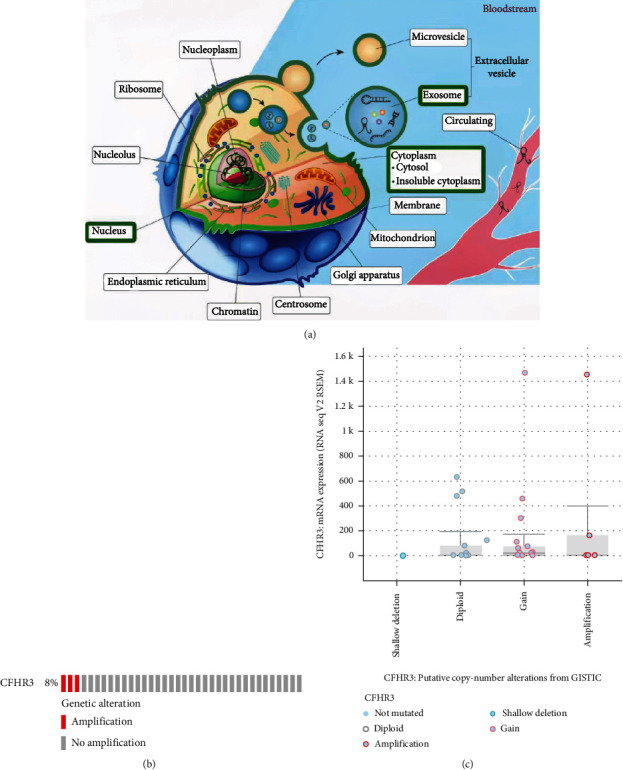
The location and mutation analysis of key gene. (a) The location of CFHR3 in the cell. (b) The mutation situation of CFHR3 in CCA. (c) An OncoPrint plot of CFHR3 in CCA.

To further understand the molecular mechanism, we undertook location and alteration analysis. The LncACTdb 3.0 database was retrieved in analyzing the CFHR3 location in the cells. As demonstrated in Figure 6(a), the key gene widely exists in the nucleus, exosome, and cytoplasm (Figure 6(a)).

cBioPortal was used to show the key gene alteration. As the OncoPrint plot shows, there was an 8% genetic alteration in the key gene in the TCGA CHOL dataset (Figure 6(b)). One diagram shows an alteration of the CFHR3 (Figure 6(c)).

## 4. Discussion

Cholangiocarcinoma is a slow-growing malignancy of the bile duct [22]. In recent years, the incidence of cholangiocarcinoma has been increasing worldwide, which makes cholangiocarcinoma a health problem of increasing concern. Current treatment options for cholangiocarcinoma are limited because early detection and surgical treatment are difficult [2]. There is an urgent need to understand the genes associated with prognosis in cholangiocarcinoma. As medical technology advances, immune checkpoint blockade (ICB) has become a new method of cancer treatment. Cholangiocarcinoma (CCA) has an abundant tumor immune microenvironment [23]. According to these findings, immune research performs a crucial role in cholangiocarcinoma treatment.

In this work, we discovered a crucial gene for predicting prognosis in CCA patients. First, we found that decreased CFHR3 expression was associated with a poor prognosis in cholangiocarcinoma patients, including overall survival and recurrence-free survival. Second, we used Cox regression analysis to show that our prognostic signature had good predictive accuracy. After 1, 2, and 3 years, low-expression CFHR3 was still a risk factor for CCA. Additionally, in the univariate and multivariate regression analysis, we found that perineural invasion could be an independent prognosis factor. A recent report has shown that an important feature of cholangiocarcinoma is peripheral nerve invasion. This may be connected with the aggressive behaviour of CCA and its poor response to treatment [24]. Therefore, CFHR3 could be a biomarker for prognosis in cholangiocarcinoma. So, the role of CFHR3 should be further investigated.

According to functional annotation, we discovered the function and pathways of CFHR3 and other coexpression genes. We analyzed the results from the GSEA analysis of the TCGA database. The drug metabolism cytochrome P450, complement and coagulation cascades, steroid hormone biosynthesis, and primary bile acid biosynthesis were all enhanced in the CFHR3 expression group. Some epidemiologic research have found that bile production and excretion may play a role in the aetiology of cholangiocarcinoma. Therefore, we identified and screened the potential coexpression genes, and the lncRNA-miRNA and lncRNA-mRNA regulation network was completed. Next, we conducted the GO and KEGG analyses for the purpose of demonstrating that these genes might participate in the complement and coagulation cascades, monocarboxylic acid metabolic process, and lipid catabolic process.

By performing the TIMER analysis, we established that there was a positive relationship between CFHR3 and the infiltration of B cells, neutrophils, and macrophages but negatively correlated with tumor purity, CD8+ T cells, and monocytes. Tumor occurrence, development, and evolution can be coordinated by immune mechanisms. B cells have been identified as a type of tumor infiltration with the adaptive immune system's ability to identify and target emerging tumor cells [25]. Recent studies indicate that the inflammatory response plays a crucial role in the microenvironment alterations of normal tissue. Neutrophils and macrophages are the important cells involved in this process [26]. Therefore, these immune cells have a close relationship with cholangiocarcinoma, which is of great significance in the treatment of CCA.

Our research has some limitations. First, our data came from the GEO and TCGA datasets, and the amount of clinical data we had was modest. Hence, larger-sample studies are needed to estimate the clinical relevance of CFHR3. Besides, in this passage, we found the difference expression of CFHR family in cholangiocarcinoma and the specific molecular mechanism should be further studied.

## 5. Conclusions

In conclusion, our findings show that reduced CFHR3 expression is associated with a poor prognosis and immune regulation in CCA patients. Further studies should be performed to study the molecular effects of CFHR3 in CCA.

## Figures and Tables

**Table 1:** The genes related with prognosis in TCGA-CHOL database.

Gene	KM	HR	HR.95 L	HR.95H	*p* value
GCNT4	0.002408	0.618419	0.453554	0.843211	0.002382
APBA2	0.006801	0.727395	0.547252	0.966837	0.028361
TTC29	0.041737	5.246029	1.065476	25.82960	0.041556
KLRB1	0.012578	0.575461	0.371508	0.891383	0.013325
NPY2R	0.000147	15.56928	1.694302	143.0692	0.015271
EIF5AL1	0.005824	5.862329	1.983363	17.32759	0.001382
TRIM31	0.037284	1.454294	1.103465	1.916662	0.007838
FAM183A	0.010867	1.629543	1.091789	2.432163	0.016860
AVPR1B	0.041278	0.445101	0.201530	0.983055	0.045259
SPDYE2	0.002466	0.306656	0.110388	0.851887	0.023362
MYBPC1	0.002620	1.759721	1.206972	2.565610	0.003305
COL4A4	0.001696	0.641426	0.454693	0.904844	0.011420
CFHR3	0.009203	1.216593	1.007963	1.468405	0.041092
GOLGA7B	0.046302	0.624614	0.411890	0.947201	0.026740
PPP1R2P1	0.024259	0.321206	0.121624	0.848299	0.021906
GRK1	0.035320	10.98407	1.455779	82.87638	0.020116
GH1	0.002271	0.005099	5.94E-05	0.437617	0.020138
C5orf46	0.015935	1.356822	1.021947	1.801428	0.034851
SERPINB13	0.007693	5.151513	1.167473	22.73123	0.030433
SLC6A14	0.035360	1.209221	1.000416	1.461608	0.049500
CRLF1	0.034175	1.656919	1.047114	2.621856	0.031038
ACR	0.024025	0.309656	0.124652	0.769232	0.011567
CST1	0.047236	1.220925	1.014977	1.468661	0.034202
PRSS35	0.001770	0.534249	0.296876	0.961419	0.036509
KRT40	0.005217	0.228907	0.053879	0.972522	0.045748
CHRM5	0.015362	0.363946	0.143565	0.922629	0.033202

**Table 2:** The baseline table of clinical information in CCA.

Characteristic	Low expression of CFHR3	High expression of CFHR3	*p* value
*n*	18	18	
Age, *n* (%)			1.000
≤65	9 (25%)	8 (22.2%)	
>65	9 (25%)	10 (27.8%)	
Gender, *n* (%)			0.315
Female	12 (33.3%)	8 (22.2%)	
Male	6 (16.7%)	10 (27.8%)	
T stage, *n* (%)			0.651
T1	8 (22.2%)	11 (30.6%)	
T2	7 (19.4%)	5 (13.9%)	
T3	3 (8.3%)	2 (5.6%)	
T4	0 (0%)	0 (0%)	
N stage, *n* (%)			1.000
N0	14 (45.2%)	12 (38.7%)	
N1	3 (9.7%)	2 (6.5%)	
M stage, *n* (%)			0.656
M0	15 (45.5%)	13 (39.4%)	
M1	2 (6.1%)	3 (9.1%)	
Pathologic stage, *n* (%)			0.543
Stage I	8 (22.2%)	11 (30.6%)	
Stage II	6 (16.7%)	3 (8.3%)	
Stage III	1 (2.8%)	0 (0%)	
Stage IV	3 (8.3%)	4 (11.1%)	
Histological type, *n* (%)			0.346
Distal	2 (5.6%)	0 (0%)	
Hilar/perihilar	1 (2.8%)	3 (8.3%)	
Intrahepatic	15 (41.7%)	15 (41.7%)	
CA19-9 level, *n* (%)			0.299
Abnormal	9 (30%)	7 (23.3%)	
Normal	5 (16.7%)	9 (30%)	
Vascular invasion, *n* (%)			0.648
No	16 (47.1%)	13 (38.2%)	
Yes	2 (5.9%)	3 (8.8%)	
Perineural invasion, *n* (%)			1.000
No	14 (42.4%)	12 (36.4%)	
Yes	4 (12.1%)	3 (9.1%)	
Age, mean ± SD	60.56 ± 15.45	65.5 ± 9.39	0.254

**Table 3:** The univariate and multivariate regression analysis of cholangiocarcinoma.

Characteristics	Total (*n*)	Univariate analysis		Multivariate analysis	
		Hazard ratio (95% CI)	*p* value	Hazard ratio (95% CI)	P value
CFHR3	36	1.265 (0.987-1.621)	0.063	1.252 (0.937-1.672)	0.128
Age	36				
≤65	17	Reference			
>65	19	1.268 (0.499-3.221)	0.617		
Gender	36				
Female	20	Reference			
Male	16	1.387 (0.544-3.534)	0.494		
T stage	36				
T1	19	Reference			
T2	12	2.612 (0.939-7.263)	0.066		
T3	5	0.986 (0.204-4.767)	0.986		
N stage	31				
N0	26	Reference			
N1	5	2.289 (0.602-8.700)	0.224		
M stage	33				
M0	28	Reference			
M1	5	1.650 (0.462-5.891)	0.440		
Pathologic stage	36				
Stage I	19	Reference			
Stage II	9	2.046 (0.646-6.476)	0.223		
Stage III	1	0.000 (0.000-Inf)	0.998		
Stage IV	7	2.279 (0.719-7.224)	0.162		
Histological type	36				
Distal	2	Reference			
Hilar/perihilar	4	130157029.581 (0.000-Inf)	0.998		
Intrahepatic	30	69806426.989 (0.000-Inf)	0.998		
CA19-9 level	30				
Abnormal	16	Reference			
Normal	14	1.003 (0.349-2.883)	0.995		
Vascular invasion	34				
No	29	Reference			
Yes	5	1.764 (0.488-6.372)	0.387		
Perineural invasion	33				
No	26	Reference			
Yes	7	4.264 (1.184-15.352)	0.026	4.871 (1.308-18.139)	0.018

## Data Availability

The publicly available datasets analyzed in this study can be found in the TCGA databases (https://portal.gdc.cancer.gov/) and GEO databases (https://www.ncbi.nlm.nih.gov/geo/).
